# Changes and Relationships Between PCB 126, PCB 169 and Macroelement Concentrations During the Technological Process of Selected High-Fat Dairy Products

**DOI:** 10.3390/molecules31162898

**Published:** 2026-08-20

**Authors:** Agata Witczak, Monika Rajkowska-Myśliwiec, Laura Przedpełska, Kamila Pokorska-Niewiada

**Affiliations:** Department of Toxicology, Dairy Technology and Food Storage, Faculty of Food Sciences and Fisheries, West Pomeranian University of Technology, 70-310 Szczecin, Poland; monika.rajkowska@zut.edu.pl (M.R.-M.); laura.przedpelska@zut.edu.pl (L.P.)

**Keywords:** macroelements, PCB 126, PCB 169, dairy processing, butter, fat blend, sweet cream

## Abstract

Dairy products are an important source of macroelements in the human diet but may also contain lipophilic contaminants such as dioxin-like polychlorinated biphenyls (dl-PCBs). This study investigated changes in the concentrations of PCB 126 and PCB 169 and selected macroelements (Ca, K, Na and Mg) during the production of butter, fat blend and cream (18% fat). Samples collected at intermediate and final stages of production were analysed. PCB congeners were determined by GC-MS, whereas macroelements were analysed by ICP-AES. Butter production increased PCB 126 and 169 concentrations on a wet weight basis, while their concentrations decreased on a lipid basis. The greatest reduction was observed during fat blend production due to the addition of sunflower oil, whereas the smallest reduction occurred during cream production. Changes in PCB 126 and 169 concentrations were accompanied by decreases in macroelement concentrations, particularly calcium. Strong negative correlations (*p* < 0.05) were found between PCB congeners and macroelements, whereas PCB 126 and PCB 169 showed strong positive correlations. These results indicate that dairy processing influences both PCB congener distribution and the macroelement composition of high-fat dairy products.

## 1. Introduction

Milk is an important component of the human diet and a valuable source of nutrients, including high-quality protein and essential amino acids [1]. Milk fat typically contains approximately 70% saturated and 30% unsaturated fatty acids [2]. Milk is also an important dietary source of essential macroelements, including calcium, potassium, sodium and magnesium [3,4], which are retained in dairy products such as cream and butter [5,6].

Besides valuable nutrients, dairy products may also contain undesirable environmental contaminants. Among these are polychlorinated biphenyls (PCBs), including dioxin-like congeners (dl-PCBs), such as PCB 126 and PCB 169, which exhibit toxicological properties similar to those of dioxins through activation of the aryl hydrocarbon receptor (AhR) [7,8,9,10].

According to the WHO toxic equivalency factor (WHO-TEF) scheme [11], PCB 126 and PCB 169 have the highest toxic equivalency factors (TEFs) among dioxin-like PCB congeners, with values of 0.1 and 0.03, respectively (Table 1). The PCB 126 and PCB 169 congeners differ not only in their TEF values (0.1 and 0.03, respectively) but also in their mode of action on the body. PCB 126 strongly activates the AhR receptor, drastically reduces liver glycogen levels, and strongly generates oxidative stress and inflammation. PCB 169, on the other hand, exhibits lower affinity for the AhR than PCB 126 and strongly accumulates in lipids, causing late-onset liver metabolic and lipid profile abnormalities. Both PCB 126 and PCB 169 drastically reduce the quality of semen in men (decrease in sperm count and motility) [12]. They inhibit the body’s immune response by inducing apoptosis (death) of spleen cells and weakening macrophages, which increases susceptibility to viral and bacterial infections [13].

Experimental studies have shown that permanent exposure in low doses to these congeners can induce oxidative stress, metabolic changes and alterations in gene expression, as well as impair liver function [14,15,16]. In addition, PCB 126 has been shown to alter the gut microbiota and promote intestinal inflammation [17,18]. Exposure to low doses of PCB 126 and PCB 169, found in food such as dairy products, does not cause acute poisoning, but due to bioaccumulation it leads to chronic destruction of the metabolic economy (diabetes, fatty liver disease), induction of systemic inflammation, neurodevelopmental disorders in offspring and a significant increase in the risk of cancer [19,20].

The main source of human exposure to dl-PCBs is contaminated food, particularly food of animal origin [21,22,23]. A global monitoring program conducted between 2017 and 2019 found dl-PCBs accounted for 63% of the total toxic equivalency (TEQ) in milk samples and 50.2% in butter samples [24]. These findings are particularly important because milk and dairy products are widely consumed and represent an important source of nutrients in the human diet. Moreover, the contribution of dl-PCB congeners to the total TEQ in milk and dairy products may reach up to 80% [25], further highlighting their importance in the toxicological assessment of these products.

**Table 1:** The physicochemical and toxicological characteristics of PCB 126 and PCB 169.

Parameter	PCB 126	PCB 169
Chemical name	3,3′,4,4′,5-Pentachlorobiphenyl	3,3′,4,4′,5,5′-Hexachlorobiphenyl
Structural formula	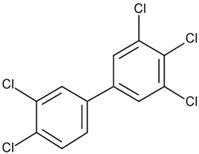	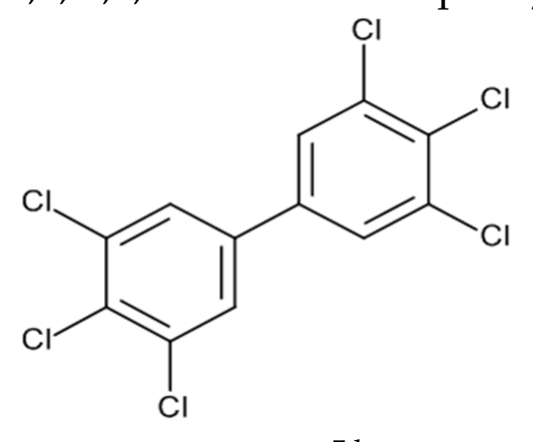
Water solubility at 25 °C, mg/L	1.03 × 10^−3 a^	3.6 × 10^−5 b^
Vapour pressure at 25 °C, mm Hg	2.96 × 10^−7 a^	4.02 ×10^−7 b^
Log *K*_ow_	6.897 ^c^	7.427 ^c^
Log BCF (zebrafish)	7.35 ^d^	7.51 ^d^
2005 WHO-TEF	0.1 ^e^	0.03 ^e^
Transfer from feed to milk (%)	40.2 ^f^	40.3 ^f^

^a^ National Toxicology Program [26], ^b^ Agency for Toxic Substances and Disease Registry [27], ^c^ Ololade et al. [28], ^d^ Mackay et al. [29], ^e^ Commission Regulation (EU) 2023/915 [11], ^f^ Amutova et al. [30].

The relevance of PCB 126 and PCB 169 is further supported by their efficient transfer from feed to milk. Reported transfer rates reached 40.2% for PCB 126 and 40.3% for PCB 169 (Table 1) [30]. This indicates that contaminated feed may represent an important pathway for the transfer of these congeners into milk and, consequently, into dairy products. Therefore, PCB 126 and PCB 169 were selected for further investigation as congeners of relevance for assessing the occurrence and potential toxicological risk of dl-PCBs in high-fat dairy products.

A number of studies have examined the effect of dairy processing on changes in PCB congener concentrations [31,32,33,34], while others have focused on changes in mineral content [35,36]. The available literature includes studies investigating correlations between PCB congeners and elemental concentrations, primarily focusing on mercury. For example, Øyan [37] notes weak positive correlations between mercury concentration and the levels of selected toxic organic contaminants (PCDD/Fs + dl-PCBs), while Renzi et al. [38] found strong positive correlations between PCB and mercury concentrations in tuna muscle. However, to the best of our knowledge, no studies have investigated whether changes in PCB congener concentrations are associated with changes in macroelement concentrations during dairy processing. Processes such as milk separation, cream pasteurization and butter churning can affect the concentrations of both macroelements and xenobiotics, such as PCBs.

Analyzing the correlations between highly toxic dioxin-like polychlorinated biphenyls (PCB 126 and PCB 169) and major milk macroelements (Ca, Mg, Na, K) has important scientific and practical implications. Because analysed PCB congeners are highly lipophilic and chemically stable (Table 1), changes in their concentrations during dairy processing are expected to result mainly from the redistribution of the lipid and aqueous phases rather than from thermal degradation.

The aim of this study was to simultaneously assess changes in nutritional value and the distribution of the most toxic PCB congeners during the production of butter, cream and fat blend. The analysis included both intermediate and final products. Our research represents the first step towards developing a model that will enable the simultaneous tracking of environmental toxins in dairy products and assessment of toxicological risks during their production while maintaining the nutritional quality of high-fat dairy products.

## 2. Results and Discussion

### 2.1. Changes in PCB 126, PCB 169 and Macroelement Concentrations During Butter Production

**Figure 1:**
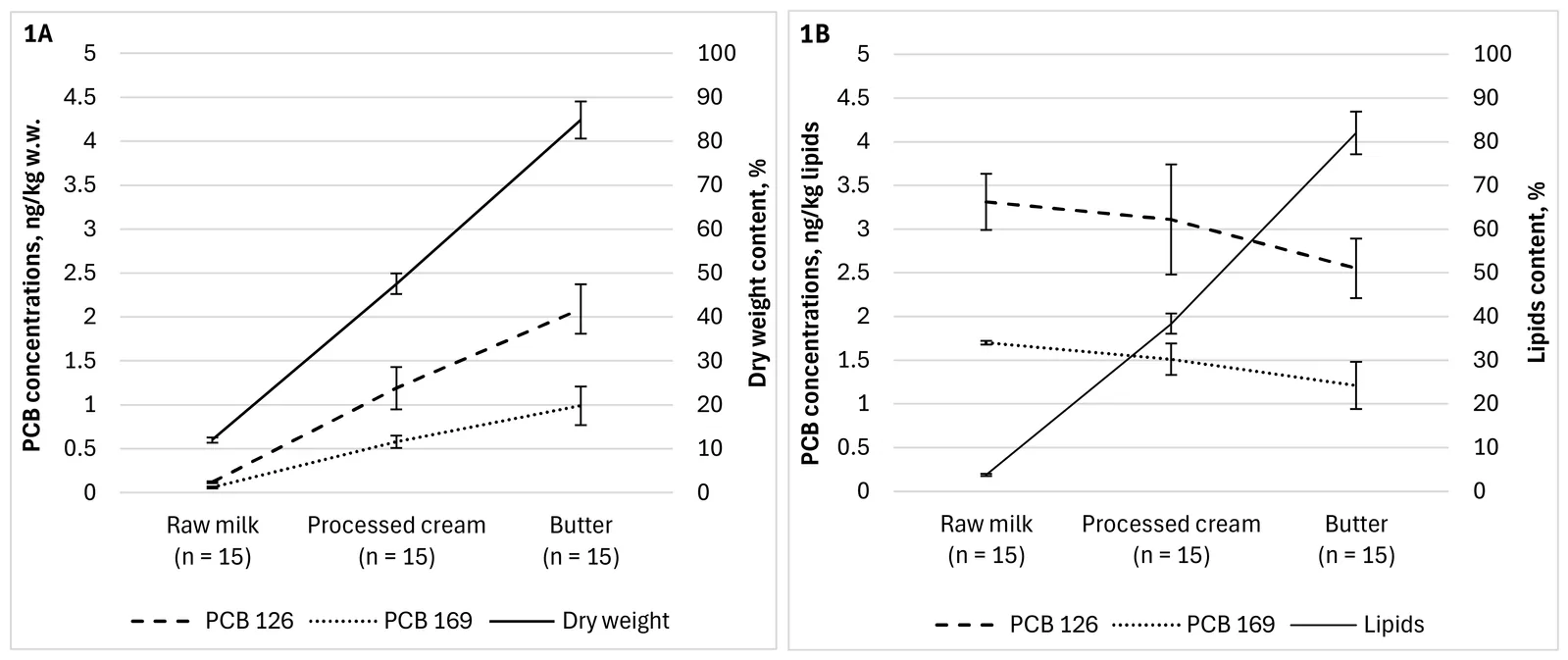
Changes in PCB 126 and PCB 169 concentrations (mean ± SD) during butter production expressed in the wet weight (**A**) and lipids (**B**). Lines connecting the data points are used only to illustrate the sequence of processing stages.

**Figure 2:**
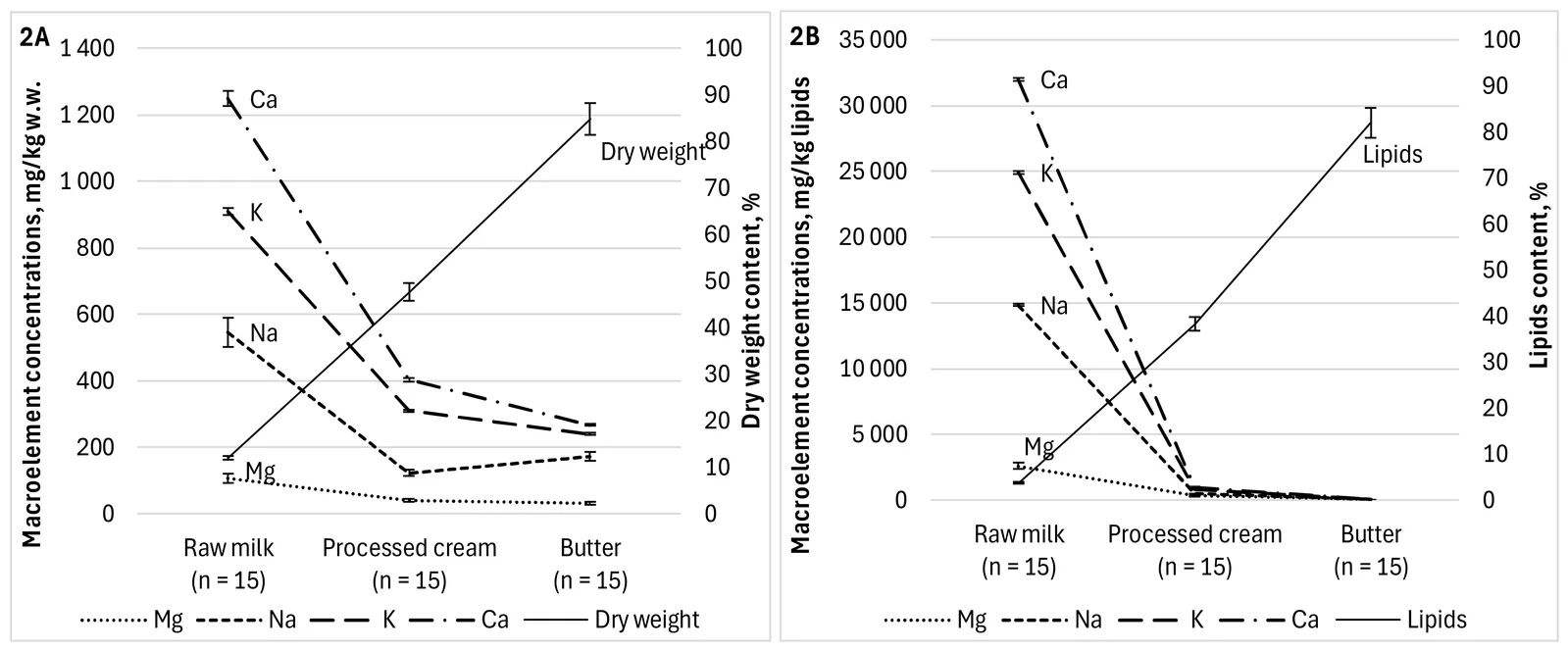
Changes in the concentrations of Mg, Na, K, Ca (mean ± SD) in the wet weight (**A**) and lipids (**B**) during butter production. Lines connecting the data points are used only to illustrate the sequence of processing stages.

**Figure 3:**
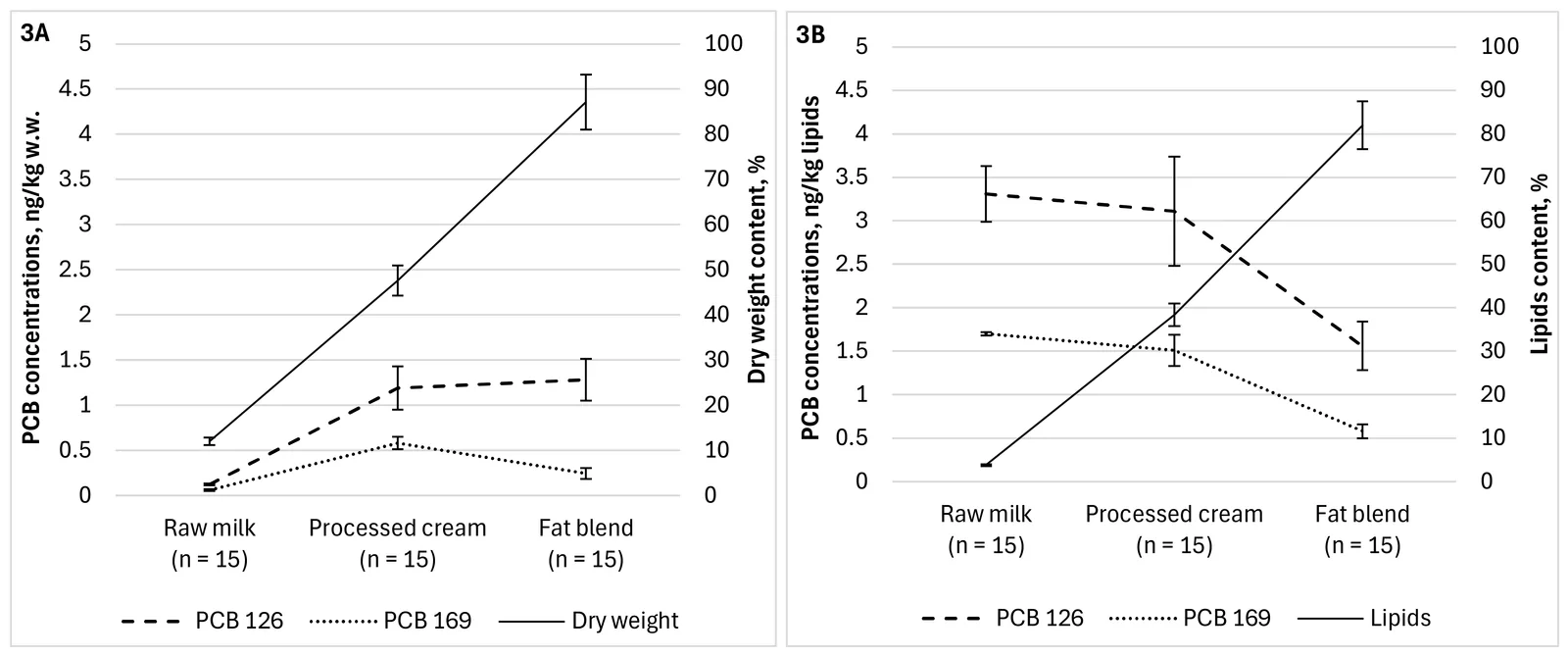
Changes in PCB 126 and PCB 169 concentrations (mean ± SD) during fat blend production expressed in the wet weight (**A**) and lipids (**B**). Lines connecting the data points are used only to illustrate the sequence of processing stages.

**Figure 4:**
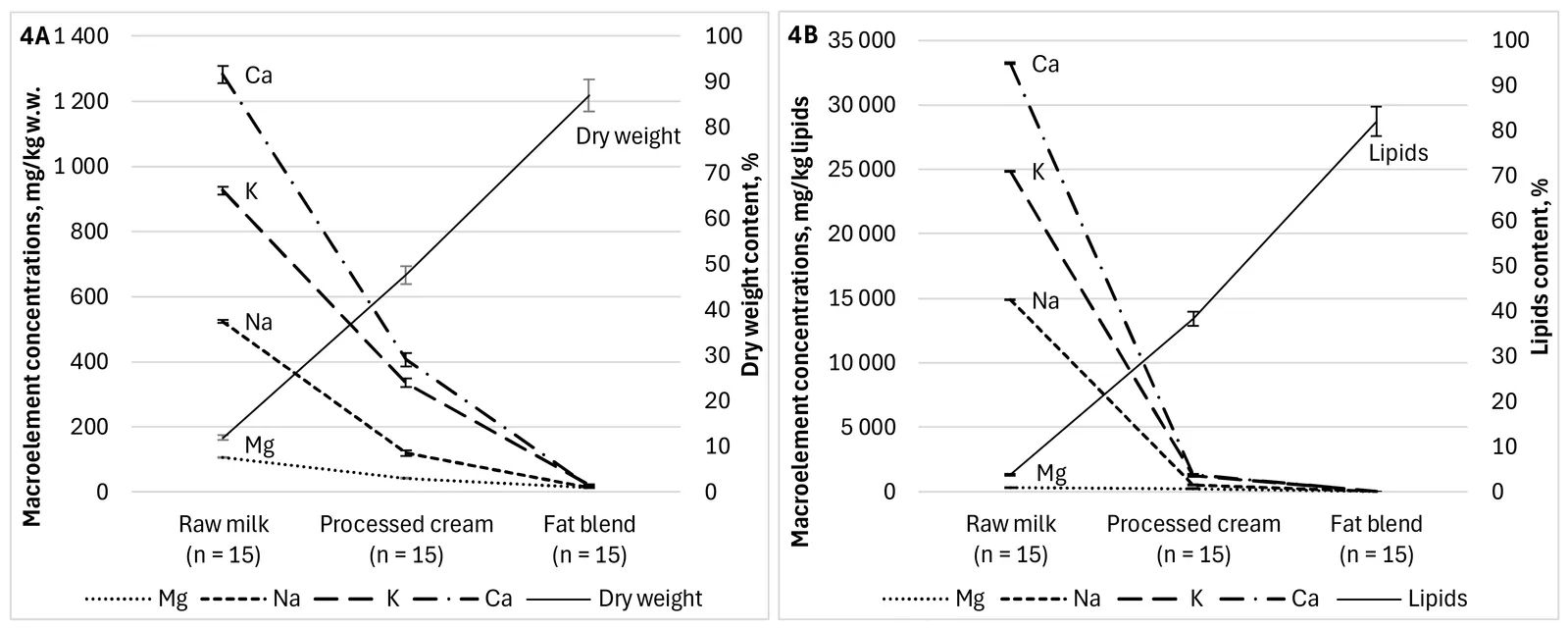
Changes in the concentrations of Mg, Na, K, Ca (mean ± SD) in the wet weight (**A**) and lipids (**B**) during fat blend production. Lines connecting the data points are used only to illustrate the sequence of processing stages.

**Figure 5:**
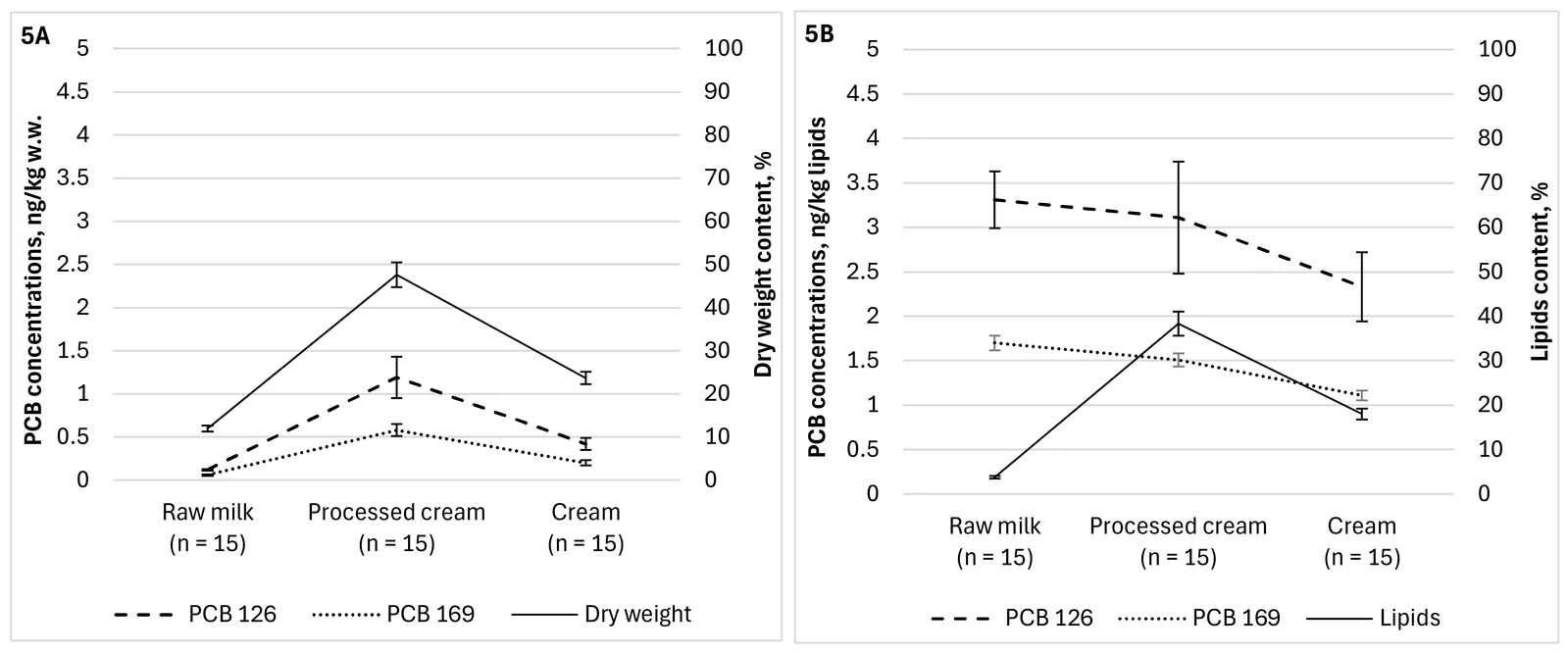
Changes in PCB 126 and PCB 169 concentrations (mean ± SD) during cream (18% fat) production expressed in the wet weight (**A**) and lipids (**B**). Lines connecting the data points are used only to illustrate the sequence of processing stages.

**Figure 6:**
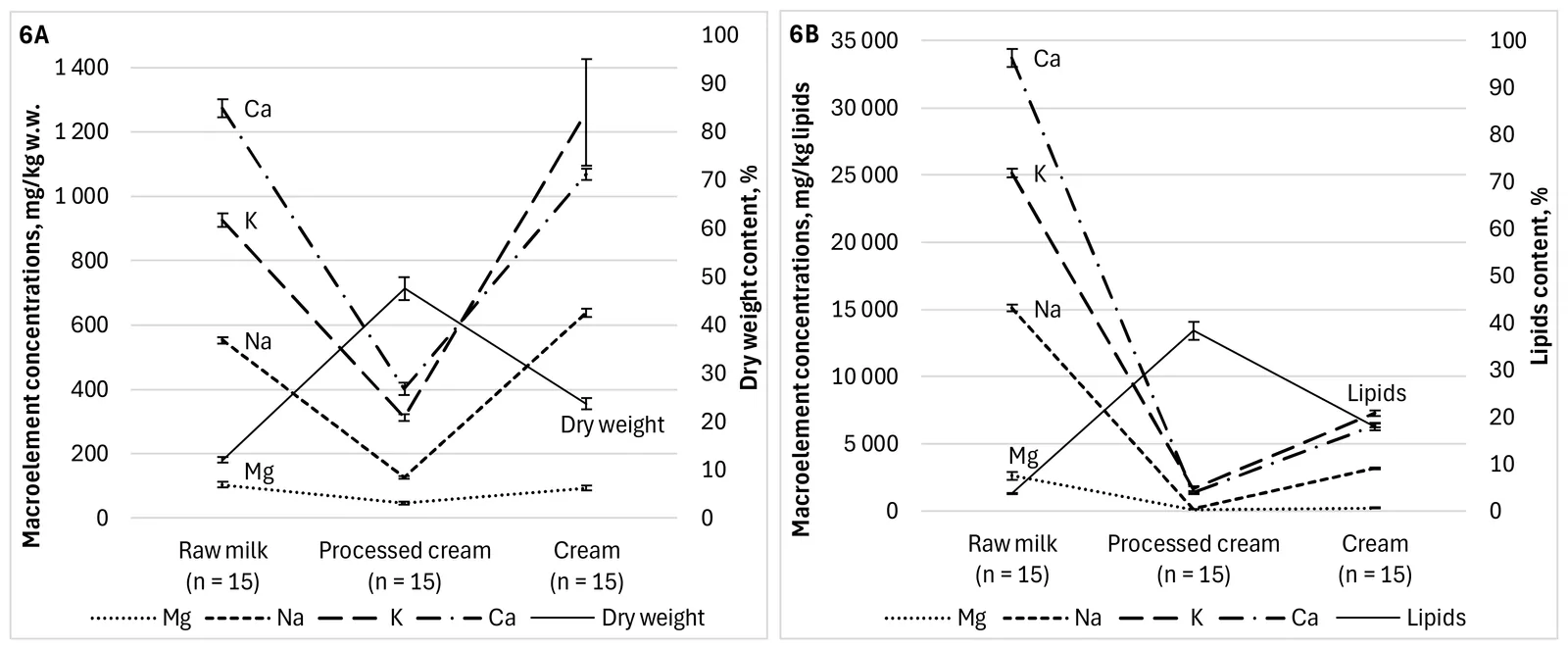
Changes in the concentrations of Mg, Na, K, Ca (mean ± SD) in the wet weight (**A**) and lipids (**B**) during cream (18% fat) production. Lines connecting the data points are used only to illustrate the sequence of processing stages.

**Figure 7:**
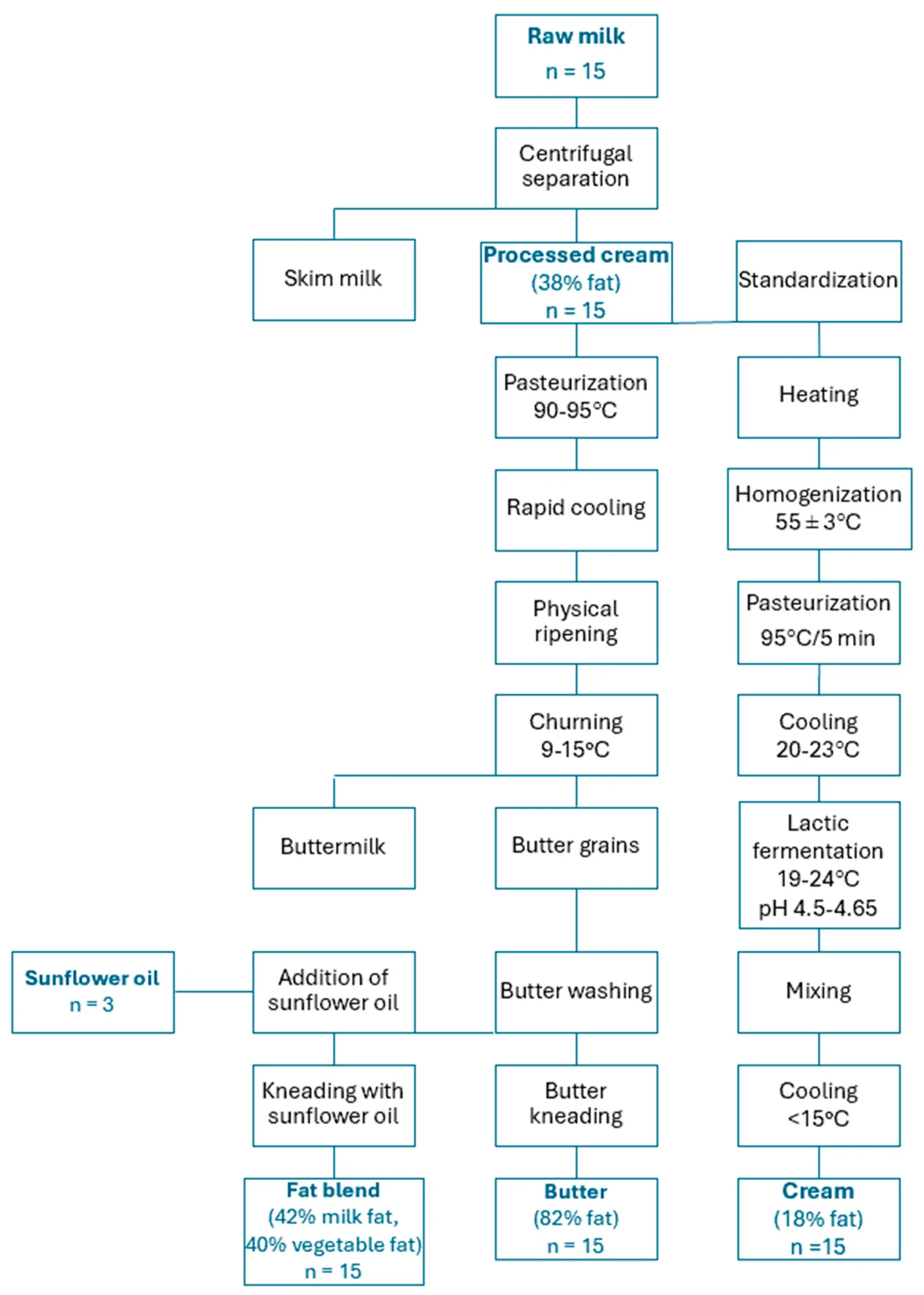
The processing scheme of the analysed dairy products. Tested products are marked in blue; n = number of experimental samples.

The experimental plan and the results of our research are presented in Figure 1, Figure 2, Figure 3, Figure 4, Figure 5, Figure 6 and Figure 7 and in Table 2, Table 3, Table 4, Table 5, Table 6 and Table 7. The production of butter from pasteurized and acidified cream involved cream ripening, churning, separation of buttermilk and a final rinse of the butter. In the present study, the process was divided into two stages due to the possibility of collecting both an intermediate product (processed cream) and the final product from the production line.

The raw milk used for the production of butter, fat blend and cream had a mean fat content of 3.75% and a dry weight content of 12.0%. The changes in fat and dry weight content occurring during butter production are shown in Figure 1. The dry weight content increased from 47.6% in the intermediate product (processed cream) to 84.9% in butter (Figure 1A). The lipid content increased from 38.3% in processed cream to 82.0% in butter (Figure 1B).

PCBs, as lipophilic compounds, are mainly found in the fat fraction of dairy products. Therefore, during the production of butter, fat blends, and cream, increasing the fat content may result in higher PCB concentrations in the final products [39,40,41,42].

The concentrations of PCB 126 and PCB 169 congeners differed significantly (*p* < 0.05) between successive stages of butter production on both a wet weight and on a lipid basis (Figure 1).

During butter production, the concentrations of PCB 126 and PCB 169 congeners increased on a wet weight basis (Figure 1A), with a significantly greater increase observed for PCB 126 (*p* < 0.05). At the intermediate stage (milk centrifugation and processed cream production), the concentrations of PCB 169 and PCB 126 congeners increased on average by 9.7- and 9.9-fold, respectively. In the final stage (butter production), the increase was 1.7- to 1.8-fold. These changes corresponded to the increase in lipid content observed in both the intermediate and final products (Figure 1B). During churning, disruption of the milk fat globule membrane and removal of the aqueous phase result in concentration of the lipid fraction, which may explain the higher PCB concentrations observed on a wet weight basis [43].

On a lipid basis, the concentrations of PCB 126 and PCB 169 decreased by 6.1% and 11.2%, respectively, during the production of processed cream (Figure 1B). Overall, compared with raw milk, the PCB concentrations in the final product decreased by 24.0% for PCB 126 and 31.0% for PCB 169. A significant proportion of lipids, including phospholipids, is transferred to the buttermilk during the churning process [44]. Hence, the subsequent removal of buttermilk may contribute to a partial reduction in PCB concentrations. This is supported by the presence of PCBs in buttermilk reported by Battisti et al. [42]. Furthermore, the reduction in PCB concentrations on a lipid basis may also be associated with the butter washing stage, during which components of the milk fat globule membrane and small fat globules may be partially removed [45].

Similar effects of dairy processing have been reported for other persistent organic pollutants. Singh and Nelapati [46] observed a reduction in HCH residues after pasteurization, boiling, and sterilization of milk, although the extent of these changes depended on the processing conditions. The lipophilic nature of POPs and their tendency to accumulate in the fat fraction may influence their distribution during milk processing [47,48]. The occurrence of organochlorine pesticides and PCBs in milk and dairy products has also been confirmed in recent monitoring studies [49,50,51].

Changes in the concentrations of PCB 126 and PCB 169 coincided with changes in macroelement concentrations during butter production. These changes affected the nutritional composition of the final products. During the first production stage, i.e., cream separation, significant decreases in macroelement concentrations were observed. On a wet weight basis, the reductions were less pronounced, with the greatest reduction reaching approximately 75% (Figure 2A, Figure 4A and Figure 6A). When the results were expressed on a lipid basis, the calcium concentration decreased by more than 96%, whereas the magnesium concentration decreased by approximately 87% (Figure 2B, Figure 4B and Figure 6B).

A key stage in the production of processed cream is milk centrifugation, during which the cream is separated from the skim milk fraction. Because most macroelements remain in the skim milk fraction, their concentrations decrease significantly in processed cream. This is particularly apparent for potassium and sodium, which are present predominantly in the serum phase of milk [36]. Calcium and magnesium differ in their distribution in milk. Calcium is an important component of colloidal calcium phosphate (CCP) associated with casein micelles, whereas magnesium is more abundant in the soluble phase and less strongly associated with the micellar fraction [52,53]. However, the association of calcium with CCP does not imply protection against losses during processing, as calcium remains in equilibrium between the colloidal and soluble phases and may redistribute during processing.

During butter production, macroelement concentrations expressed on a lipid basis decreased significantly (*p* < 0.05), with the greatest reduction observed for calcium (Figure 2B). On a wet weight basis, Bilandžić et al. [6] reported that the calcium concentration in butter was approximately 10-fold lower than that in milk. These changes may be explained by the separation of the skim milk fraction during churning, as most macroelements remain in this fraction [36,54].

### 2.2. Changes in PCB 126, PCB 169 and Macroelement Concentrations During Fat Blend Production

Among the three dairy products analysed, fat blend production resulted in the greatest reduction in PCB concentrations. These reductions may be associated with the lower proportion of milk fat in the final product, as part of the milk fat was replaced with vegetable fat (sunflower oil). Overall, compared with raw milk, the concentrations of PCB 126 and PCB 169 in the final product were reduced by 55% and 72%, respectively, on a wet weight basis (Figure 3A). The corresponding reductions, expressed on a lipid basis, were 49.8% for PCB 126 and 61.6% for PCB 169 (Figure 3B).

In the sunflower oil used for fat blend production, PCB 126 was detected at a concentration of 0.12 ± 0.02 ng/kg lipids, whereas PCB 169 was below the limit of detection (<LOD). The concentrations of both congeners were considerably lower than those found in dairy products of animal origin. Similar findings were reported by Saktrakulkla et al. [55], who did not detect any of the 205 PCB congeners analysed in vegetable oil and margarine samples. Other studies also reported lower concentrations of dl-PCBs in vegetable oils and fats than in products of animal origin [56,57]. This may be due to industrial refining processes, particularly deodorisation, which can almost completely remove these contaminants [58,59]. Therefore, the addition of vegetable oil to animal fat may reduce PCB concentrations in the final product. This may be related to the lower concentrations of these contaminants in vegetable oils than in animal fats [60,61].

Changes in macroelement concentrations during fat blend production followed a pattern similar to that observed during butter production. On a wet weight basis, the reductions in macroelement concentrations were considerably smaller (Figure 4A). On a lipid basis, the smallest reduction was observed for magnesium, whereas the greatest was observed for calcium (Figure 4B).

The addition of vegetable fat to the fat blend had no significant effect on the concentrations of the analysed macroelements. Giacomino et al. [62] reported calcium, potassium, magnesium and sodium concentrations in sunflower oil of 8.25–12.3 mg/kg, <0.02–8.26 mg/kg, 1.55–2.30 mg/kg and 0.24–1.91 mg/kg, respectively. The lower reductions in macroelement concentrations compared with butter may reflect differences in the composition of the fat blend and the technological process.

### 2.3. Changes in PCB 126, PCB 169 and Macroelement Concentrations During Cream (18% Fat) Production

The production of cream (18% fat) from processed cream included homogenisation and pasteurisation. During cream production, the decrease in lipid content compared with processed cream was accompanied by a decrease in dry weight content.

On a wet weight basis, the concentrations of PCB 126 and PCB 169 decreased approximately twofold throughout the production of 18% cream (Figure 5A). These reductions may be related to the lower lipid content of the final product (18%) compared with processed cream (38%).

On a lipid basis, cream showed the smallest reduction in PCB 126 and 169 concentrations among the final products analysed (Figure 5B). The concentrations of PCB 126 and PCB 169 decreased by 25.1% and 26.5%, respectively. Thermal processing does not generally result in substantial elimination of PCB congeners [33,40] and may even affect the content of some PCB congeners [32].

During the production of homogenised, pasteurised cream, the concentrations of all analysed macroelements decreased significantly in processed cream and then increased in the final product.

On a wet weight basis, these changes were less pronounced (Figure 6A). The final 18% cream contained higher Na and K concentrations than the raw milk.

On a lipid basis, a similar decrease followed by an increase was observed (Figure 6B). As discussed above, the initial reduction in macroelement concentrations during processed cream production may be explained by their predominant distribution in the aqueous phase.

Cream processing may involve the addition of calcium salts to improve product stability and whipping properties [63], which may contribute to the calcium concentrations of the final product. In addition, emulsifiers and stabilizers containing sodium compounds, such as sodium alginate, may also be used in cream production [64], which may partly explain the higher sodium concentrations [36].

### 2.4. Correlations Between Changes in Lipid Content, Macroelement Concentrations and PCB Congeners

Analysis of the relationships between changes in lipid content and macroelement concentrations revealed strong negative correlations, with correlation coefficients ranging from −0.749 to −0.977 (*p* < 0.05). The strongest negative correlations were observed for calcium and potassium. This may be explained by the fact that these macroelements are mainly present in the aqueous phase, and their concentrations decrease as lipid content increases.

On a wet weight basis, particularly in butter and cream, strong positive correlations were observed between changes in lipid content and the concentrations of PCB 126 and PCB 169 congeners (r = 0.80–0.99; *p* < 0.05). This relationship may reflect the removal of the aqueous phase during processing, resulting in a higher proportion of the lipid fraction and, consequently, higher PCB concentrations on a wet weight basis.

On a lipid basis, strong negative correlations were observed for butter and fat blend (Table 2). Although PCB congeners accumulate in lipids, increasing lipid content may result in dilution of their concentrations, as also observed during the analysis of changes in PCB 126 and PCB 169 concentrations (Figure 1B and Figure 3B). In contrast, only weak correlations were observed for PCB 126 and PCB 169 in cream on a lipid basis (r = −0.22 and −0.09, respectively; *p* < 0.05). This may partly reflect the lower lipid content of the final product compared with the processed cream.

### 2.5. Correlations Between PCB 126, PCB 169 and Macroelements

Strong negative correlations were observed between changes in PCB 126, PCB 169 and macroelement concentrations during the production of the analysed dairy products (Table 3, Table 4, Table 5, Table 6 and Table 7). In general, increases in PCB 126 and PCB 169 concentrations were accompanied by decreases in macroelement concentrations (Table 3). These correlations may reflect the redistribution of PCB congeners and macroelements between the lipid and aqueous phases during processing rather than a direct physicochemical interaction between them. It should be emphasized that the observed correlation is more likely associated with differences in the composition of dairy products and the technological processes used, and not a direct effect of macroelements on the occurrence of the analysed PCB congeners.

In butter, moderate to strong negative correlations were observed between both PCB congeners and all analysed macroelements on a wet weight basis, whereas strong to very strong negative correlations were found on a lipid basis (Table 3). The strongest negative correlations were observed between PCB 169 and the macroelements on a lipid basis, with correlation coefficients ranging from −0.96 to −0.97 (*p* < 0.05). In milk, calcium may interact with components of the phospholipid-rich milk fat globule membrane (MFGM) [65]. For example, during butter manufacture, churning disrupts the MFGM, releasing membrane fragments and polar lipids into the aqueous phase, including buttermilk and butter serum [45,66]. Subsequent washing and working remove part of this aqueous phase, resulting in a final product that is roughly 82% fat and contains relatively low concentrations of Ca.

In the fat blend, very strong negative correlations were observed between PCB 126 and PCB 169 and all analysed macroelements (Table 4). The strongest correlations were found for PCB 126 on a wet weight basis (r = −0.96 to −0.97; *p* < 0.05) and for PCB 169 on a lipid basis (r = −0.98 to −0.99; *p* < 0.05).

In cream, the correlation pattern differed from that observed in butter and fat blend (Table 5).

On a wet weight basis, PCB 126 showed strong negative correlations with Mg and Ca, whereas its correlations with Na and K were weak. In contrast, PCB 169 showed only weak correlations with all analysed macroelements. On a lipid basis, PCB 126 was strongly negatively correlated with all analysed macroelements, whereas the correlations for PCB 169 were weaker, with weak correlations observed for Na and K (*p* < 0.05). The weaker correlations observed in cream may be related to the approximately one-half lower lipid content in the final product compared with processed cream.

PCB congeners accumulate in the lipid fraction, whereas macroelements are mainly present in the aqueous phase. As a result, higher PCB concentrations were associated with lower macroelement concentrations. Strong positive correlations were also observed between the analysed macroelements. In addition, PCB 126 and PCB 169 were strongly positively correlated (Table 5), suggesting similar behaviour during dairy processing.

The results indicate that dairy processing mainly affects the distribution of PCB congeners by changing the proportions of the lipid and aqueous phases, rather than by directly removing these contaminants. Therefore, PCB congener concentrations in dairy products depend primarily on their concentrations in raw milk, whereas technological processing mainly influences their distribution within dairy products.

### 2.6. Limitations of the Study

This work covers a selected series of analyses. The focus was on the most toxic compounds, PCB 126 and PCB 169, classified by IARC [67] as carcinogenic (Group 1). As indicated earlier, our research represents the first step towards developing an advanced model that will enable the tracking of environmental toxins in dairy products and assessment of toxicological risks associated with their presence during production. Further research will therefore be continued.

## 3. Materials and Methods

### 3.1. Reagents and Reference Materials

All the reagents used in the analyses were of high purity (Suprapur^®^, Merck KGaA, Darmstadt, Germany).

The following standard solutions were used for PCB analysis.

- 3,3′,4,4′,5-pentachlorobiphenyl (PCB 126, CAS No. 57465-28-8) and 3,3′,4,4′,5,5′-hexachlorobiphenyl (PCB 169, CAS No. 32774-16-6; LGC Standards, Wesel, Germany);
- ^13^C_12_-labeled PCB Mixture-A (EC-4938, Cambridge Isotope Laboratories, Inc.) consisting of: 3,3′,4,4′-tetrachlorobiphenyl (PCB 77); 3,4,4′,5-tetrachlorobiphenyl (PCB 81); 2′,3,4,4′,5-pentachlorobiphenyl (PCB 123); 3,3′,4,4′,5-pentachlorobiphenyl (PCB 126); 3,3′,4,4′,5,5′-heksachlorobiphenyl (PCB 169); 2,2′,3,4,4′,5,5′-heptachlorobiphenyl (PCB 189);
- Pesticide Surrogate Spike Mix (CRM48460, Cerilliant Corporation, Round Rock, TX, USA), containing decachlorobiphenyl (PCB 209) and 2,4,5,6-tetrachloro-m-xylene, used as surrogate standards for recovery control.
- certified reference material: BCR 450 (PCBs in natural milk powder, Community Bureau of Reference, Joint Research Centre, Ispra, Italy).

The following standard solutions were used for macroelement content analysis:

- magnesium standard solution—traceable to SRM from NIST: Mg(NO_3_)_2_ in HNO_3_ 0.5 mol/L, 1000 mg/L Mg (Certipur^®^, Merck KGaA, Darmstadt, Germany);
- calcium standard solution—traceable to SRM from NIST: Ca(NO_3_)_2_ in HNO_3_ 0.5 mol/L, 1000 mg/L Ca (Certipur^®^, Merck KGaA, Darmstadt, Germany);
- potassium standard solution—traceable to SRM from NIST: KNO_3_ in HNO_3_ 0.5 mol/L, 1000 mg/L K (Certipur^®^, Merck KGaA, Darmstadt, Germany);
- sodium standard solution—traceable to SRM from NIST: NaNO_3_ in HNO_3_ 0.5 mol/L, 1000 mg/L Na (Certipur^®^, Merck KGaA, Darmstadt, Germany);
- certified reference material: Skim milk powder ERM-BD151 (European Reference Material, Geel, Belgium).

### 3.2. Study Materials

Raw material (raw milk), intermediate products (processed cream) and final products (butter, fat blend and cream) were collected directly from industrial production lines at a dairy plant in Poland (Figure 7). Three independent production batches were included in the study, and five samples (1 L each) were collected from each batch at each technological stage (n = 15 per stage). Each experimental sample was analysed in five analytical replicates.

Because sunflower oil is one of the ingredients of the fat blend, it was also included in the analysis. Three sunflower oil samples were collected, one from each production batch (n = 3).

Samples of raw milk and cream were collected during production; these were placed into clean, solvent-rinsed (HPLC-grade acetone) glass jars, each with a capacity of 1 L. The samples were then transported to the laboratory under refrigerated conditions at a controlled temperature (5 ± 1 °C). The levels of dry weight, fat, PCB 126 and PCB 169 were determined, as well as the macroelements Ca, Na, K and Mg.

### 3.3. Determination of Lipid and Dry Weight Content

The fat content was determined by extraction with n-hexane in a Soxhlet apparatus for eight hours. The dry weight content was determined gravimetrically in accordance with ISO 6731:2010 [68]. The test samples were dried to constant weight in a laboratory oven at 102 ± 2 °C.

Dry matter and lipid content were determined in five analytical replicates and are presented as mean ± SD.

### 3.4. Determination of Macroelements Content

Samples of butter and fat blend (0.5 ± 0.02 g) were weighed into Teflon vessels, and 5 mL of 65% HNO_3_ (Suprapur, Merck KGaA, Darmstadt, Germany) was added. Samples were digested in a microwave digestion system (MDS 2000, CEM Corp., Matthews, NC, USA) using a five-step digestion program. The first two stages were performed at 85% microwave power, followed by two stages at 100% power, with pressure gradually increasing from 20 to 150 PSI. After digestion, the samples were cooled, filtered into 25 mL volumetric flasks and diluted to volume with ultrapure water (0.05 μS/cm; Barnstead™GenPure™Pro, Thermo Scientific, Langenselbold, Germany).

The concentrations of Ca, K, Mg and Na were determined by inductively coupled plasma atomic emission spectrometry (ICP-AES; Jobin Yvon JY-24). The instrument was operated at a power of 1000 W and a frequency of 40.68 MHz using argon as both the plasma gas (12 L/min) and nebulizer gas (0.7 L/min). The analytical wavelengths were 279.5 nm for Mg, 393.4 nm for Ca, 589.0 nm for Na and 766.5 nm for K.

#### Macroelements Analysis Quality Control

Method accuracy and precision were verified using the certified reference material Skim Milk Powder ERM-BD151 (European Reference Material, Geel, Belgium).

Calibration was performed using standard solutions. Calibration curves were evaluated by least-squares linear regression, and the slope, intercept, and coefficient of determination (R^2^) were determined. Working standard solutions were prepared in volumetric flasks using 15% HNO_3_ in ultrapure Milli-Q water (Merck, Darmstadt, Germany) by serial volume/volume dilution of the corresponding single-element standard solutions. Calibration curves with a coefficient of determination (R^2^) ≥ 0.995 were considered acceptable.

The method was validated by determining the limit of detection (LOD), limit of quantification (LOQ), recovery and coefficient of variation (CV) (Table 6). 

The LOD and LOQ were calculated using the standard deviation (SD) of the intercept and the slope (S) of the calibration curve, according to the following equations [69]:

(1) $$LOD=3.3\;\times \frac{SD}{S}$$

(2) $$LOQ=10\times \frac{SD}{S}$$

### 3.5. Determination of PCB Congener Content

#### 3.5.1. Sample Preparation

Upon delivery to the laboratory, raw milk samples were freeze-dried in a LyoLab 3000 freeze dryer (Heto-Holten A/S, Allerød, Denmark) at −60 °C for 72 h. The freeze-dried samples were then stored at −18 °C in tightly closed containers until analysis.

Cream, butter and fat blend samples were stored at −18 °C until analysis. Prior to analysis, the samples were thawed at 5 °C and homogenized. Because of their high lipid content, the samples were dried by homogenization with anhydrous sodium sulphate. Samples weighing 10 ± 0.03 g were taken for analysis. All analyses were performed in five analytical replicates.

#### 3.5.2. Extraction and GC-MS Analysis

PCB congeners were extracted using n-hexane/acetone (3:1, *v*/*v*) according to the validated method described by Witczak et al. [70]. This solvent mixture has also been used for PCB extraction from other complex matrices [71,72]. The samples were extracted in a Soxhlet apparatus for 8 h using 120 mL of n-hexane/acetone (3:1, *v*/*v*). The extracts were concentrated to 2 mL and purified with 8 mL of fuming H_2_SO_4_ (7% SO_3_ in concentrated H_2_SO_4_), followed by further purification on a 1 g bed of basic alumina. PCBs were eluted sequentially with 14 mL of 2% DCM in n-hexane and 17 mL of 50% DCM in n-hexane. The purified extracts were concentrated under a stream of nitrogen to 0.1 mL. Method performance was verified by recovery tests and analysis of the certified reference material BCR 450. The extracts were analysed by gas chromatography–mass spectrometry (GC–MS; HP 6890/5973) using an HP-5 MS capillary column (60 m × 0.25 mm × 0.25 μm). Each analytical replicate (n = 75) was injected into the GC–MS system in triplicate.

Quantitative analysis was performed in selected ion monitoring (SIM) mode using the molecular ions *m*/*z* 326 for PCB 126 and *m*/*z* 360 for PCB 169. The confirmation ions were *m*/*z* 324 and 254 for PCB 126, and *m*/*z* 362 and 290 for PCB 169.

The following chromatographic conditions were applied: oven temperature program: 130 °C for 0.5 min, increased at 7 °C/min to 200 °C and held for 5 min, then increased at 4 °C/min to 280 °C and held for 10 min, followed by a post-run at 290 °C for 5 min. The injection volume was 2 μL. Helium (6.0) was used as the carrier gas at a flow rate of 1.1 mL/min. The total run time was 50 min [70].

#### 3.5.3. Quality Control of PCB Congener Analysis

Samples were spiked with 50 µL of ^13^C_12_-labelled PCB Mixture-A (EC-4938, Cambridge Isotope Laboratories, Inc., Tewksbury, MA, USA; 120 ppb) as internal standards and with 100 µL of Pesticide Surrogate Spike Mix (CRM48460, Cerilliant Corporation, Round Rock, TX, USA; 80 ng/mL) containing PCB 209 as the surrogate standard for recovery control.

The recoveries obtained for PCB 169 and PCB 126 were 79.59% (CV, 4.57%) and 87.40% (CV, 6.36%), respectively. The recovery of PCB 209 ranged from 84.10 ± 1.38% to 98.32 ± 1.15%.

Method accuracy was verified using the certified reference material BCR 450 (PCBs in natural milk powder, Community Bureau of Reference, Joint Research Centre, Ispra, Italy), which was analysed with each analytical batch.

Calibration curves for PCB 126 and PCB 169 were prepared in n-hexane in the range of 0.5–120 ppb for fortified samples and 0.5–50 ppb for real samples.

The limit of detection (LOD) and limit of quantification (LOQ) were estimated based on signal-to-noise (S/N) ratios of 3.3 and 10, respectively [69], according to Equations (1) and (2). The key validation parameters of the method are summarized in Table 7.

### 3.6. Statistical Analysis

Pearson correlation coefficients were calculated using 45 observations for each production process, covering three technological stages. At each stage, 15 experimental samples were included (three independent production batches × five samples per batch). Analytical replicates were averaged before statistical analysis. Correlation strength was classified according to Pearson’s correlation coefficient (r): |r| < 0.7, weak to moderate correlation; |r| ≥ 0.7, strong correlation, with *p* < 0.05 considered statistically significant. Data normality was assessed using the Kolmogorov–Smirnov test, and homogeneity of variances using Levene’s test (*p* > 0.05). Tukey’s HSD test was used as a post-hoc test following ANOVA (α = 0.05). All analyses were performed using Statistica 13.0 (StatSoft, Kraków, Poland).

## 4. Conclusions

The technological processes used during the production of butter, fat blends, and cream influenced the concentrations of PCB congeners and macroelements in the analysed products. Changes observed during individual processing stages were strongly associated with the redistribution of fat and aqueous fractions.

Butter and cream production resulted in increased PCB 126 and PCB 169 concentrations on a wet weight basis, mainly due to the higher fat content of the final products. In contrast, the addition of vegetable fat during fat blend production reduced PCB concentrations in the lipid fraction.

The production processes also affected macroelement levels, particularly calcium and potassium, which were largely associated with the aqueous fraction. Strong negative correlations were observed between PCB congeners and macroelement concentrations, reflecting differences in their distribution between lipid and aqueous phases. The obtained results indicate that dairy processing stages may significantly influence both contaminant distribution and the nutritional composition of final dairy products.

The results of our study may be useful for dairy manufacturers and food safety monitoring. This research can be the first step towards developing a model for tracking environmental contaminants and macroelements.

## Figures and Tables

**Table 2:** Correlation coefficients (r; *p* < 0.05) between changes in the levels of selected macroelements and PCB congeners during dairy processing.

Dairy Product		Mg	Ca	Na	K	PCB 126	PCB 169
Butter	in wet weight	−0.863	−0.893	−0.758	−0.878	0.98	0.99
	in lipids	−0.838	−0.841	−0.834	−0.841	−0.92	−0.95
Fat blend	in wet weight	−0.890	−0.940	−0.910	−0.940	0.86	0.28 *
	in lipids	−0.840	−0.844	−0.839	−0.844	−0.93	−0.95
Cream (18% fat)	in wet weight	−0.927	−0.977	−0.850	−0.749	0.93	0.80
	in lipids	−0.899	−0.885	−0.915	−0.926	−0.09 *	−0.22 *

* weak correlation (|r| < 0.7); *p* < 0.05.

**Table 3:** Correlation coefficients (r; *p* < 0.05) between changes in the concentrations of PCB congeners and macroelements during the production of butter.

	r (in wet weight) (*p* < 0.05)					
	PCB 126	PCB 169	Mg	Ca	Na	K
PCB 126		0.94	−0.88	−0.90	−0.79	−0.89
PCB 169			−0.81	−0.85	−0.70	−0.83
Mg				1.00	0.98	1.00
Ca					0.97	1.00
Na						0.98
	r (in lipids) (*p* < 0.05)					
	PCB 126	PCB 169	Mg	Ca	Na	K
PCB 126		0.95	−0.90	−0.90	−0.89	−0.90
PCB 169			−0.97	−0.96	−0.97	−0.96
Mg				0.99	0.99	0.99
Ca					0.99	0.99
Na						0.99

**Table 4:** Correlation coefficients (r; *p* < 0.05) between changes in the concentrations of PCB congeners and macroelements during the production of fat blend.

	r (in wet weight) (*p* < 0.05)					
	PCB 126	PCB 169	Mg	Ca	Na	K
PCB 126		0.82	−0.97	−0.96	−0.97	−0.96
PCB 169			−0.87	−0.92	−0.88	−0.92
Mg				0.99	1.00	0.99
Ca					1.00	1.00
Na						1.00
	r (in lipids) (*p* < 0.05)					
	PCB 126	PCB 169	Mg	Ca	Na	K
PCB 126		0.94	−0.93	−0.93	−0.93	−0.93
PCB 169			−0.99	−0.99	−0.98	−0.98
Mg				0.99	0.99	0.99
Ca					0.99	0.99
Na						0.99

**Table 5:** Correlation coefficients (r; *p* < 0.05) between changes in the concentrations of PCB congeners and macroelements during the production of cream (18% fat).

	r (in wet weight) (*p* < 0.05)					
	PCB 126	PCB 169	Mg	Ca	Na	K
PCB 126		0.90	−0.74	−0.84	−0.62 *	−0.48 *
PCB 169			−0.55 *	−0.67 *	−0.41 *	−0.26 *
Mg				0.99	0.99	0.94
Ca					0.94	0.87
Na						0.99
	r (in lipids) (*p* < 0.05)					
	PCB 126	PCB 169	Mg	Ca	Na	K
PCB 126		0.62 *	−0.96	−0.96	−0.97	−0.97
PCB 169			−0.70	−0.71	−0.67 *	−0.66 *
Mg				0.99	0.99	0.99
Ca					0.99	0.99
Na						0.99

* weak correlation (|r| < 0.7); *p* < 0.05.

**Table 6:** Validation parameters for macroelement determination.

Macroelement	LOD (µg/kg)	LOQ (µg/kg)	Recovery (%)	CV (%)
Mg	12.7	40.2	95.5	4.1
Na	15.8	48.2	98.9	2.5
K	18.1	58.9	96.2	3.8
Ca	16.6	51.2	97.4	3.9

LOD—limit of detection; LOQ—limit of quantification; CV—coefficient of variation.

**Table 7:** Validation parameters for PCB 126 and 169 determination.

Parameter	LOD (ng/kg)	LOQ (ng/kg)	Recovery (%)	CV (%)
PCB 126	0.025	0.085	87.40	6.36
PCB 169	0.030	0.120	79.59	4.57

LOD—limit of detection; LOQ—limit of quantification; CV—coefficient of variation.

## Data Availability

Data are contained within the article.
